# Crossword puzzle as a learning tool to enhance learning about anticoagulant therapeutics

**DOI:** 10.1186/s12909-022-03348-0

**Published:** 2022-04-11

**Authors:** Ghada Bawazeer, Ibrahim Sales, Huda Albogami, Ahmed Aldemerdash, Mansour Mahmoud, Majidah A. Aljohani, Abdullah Alhammad

**Affiliations:** 1 Department of Clinical Pharmacy, College of Pharmacy, King Saud University, P.O. Box 89885, Riyadh, 11692 Saudi Arabia; 2 Pharmaceutical Care Services, Ministry of National Guard Health Affairs, Riyadh, Saudi Arabia; 3 Department of Clinical and Hospital Pharmacy, College of Pharmacy, Taibah University, Al-Madinah Al-Munawarah, Saudi Arabia; 4 Pharmaceutical Care Division, King Saud Medical City, Riyadh, Saudi Arabia

**Keywords:** Crossword game, Pharmacy, Anticoagulation, Therapeutics, Learning style, Active learning

## Abstract

**Background:**

Educational games make the learning process more enjoyable, fun, and create a competitive classroom environment that can positively affect learning. The purpose of this study was to evaluate pharmacy students’ perceptions of crossword puzzles (CWPs) as a learning tool in the pharmacotherapy cardiovascular module focusing on anticoagulants’ therapeutics and assessing if students’ preference of learning style influenced their perception.

**Methods:**

Clues for the puzzle were developed, validated, and piloted by course faculty. A free internet puzzle generator was used to create puzzles with 10 to 20 clues. Students were given 30 min to solve the puzzle following six hours of didactic lectures about the topic. An 8-item survey instrument and Pharmacists’ Inventory of Learning Styles (PILS) questionnaire were administered to examine students’ perceptions of the game and their learning style preference, respectively.

**Results:**

Two hundred sixty-seven students participated in the activity from both undergraduate programs (BPharm and PharmD) over three consecutive course offerings. Most students expressed favorable perceptions of the puzzle. Female and BPharm students had significantly more favorable perceptions than male and PharmD students on several perception items. The dominant preferred learning style (PLS) was converger (35.6%), followed by assimilator (25.3%), while 15.1% had mixed learning styles. The study did not find a significant association between PLS and students’ perceptions toward the CWP.

**Conclusions:**

The CWP game presented an innovative, creative, and easy active learning tool to enhance information recall, retention, and class engagement while accommodating all learning style preferences.

## Background

Old habits are hard to break. Didactic lectures continue to resemble the school days of Charlie Brown and his schoolteacher in the classroom. The COVID-19 pandemic has further complicated the effective delivery of didactic lectures without visual confirmation that students are actively participating and understanding the information. Such a barrier has led to a lack of learner engagement, as evident by an increase in absenteeism in pharmacy school lectures, students underestimating the importance of lectures, and a corresponding decrease in performance, particularly in therapeutics courses [1, 2]. Contrarily, students who are stimulated by effective learning methods are more likely to attend lectures than their colleagues [3]. On average, the first 10 min of a lecture are associated with the peak of student attention and highest retention of information [4–6]. Student interest declines from that point and is barely salvaged in the last 5 to 10 min. In totality, students are disengaged for approximately 75% of the lecture. This is not surprising since millennials have been weaned upon technological advances and various means of communication and entertainment, and the absence of consistent stimulation make things no longer intriguing [7, 8].

Evidence supports the positive impact of using innovative educational tools to benefit the teaching and learning process for health professions, and pharmacy education is no exception [9]. The Accreditation Council for Pharmacy Education (ACPE) placed high emphasis in “Standard 10: Curriculum Design, Delivery, and Oversight” on student engagement through teaching and learning activities that promote self-directed learning and accommodate the diverse learning needs of students [10]. Gleason and colleagues categorized classroom-based active learning methods into five types: Cooperative Learning, Problem-Based Learning, Team-Based Learning, Case-Based Learning, and Ability-Based Education and Assessment-as-Learning [11]. Stewart et al. surveyed 114 US colleges and schools of pharmacy faculty members to evaluate the extent and the promoting factors for using active learning techniques [12]. 87% of the faculty members reported using active-learning activities such as case-based learning (71%), deliberative discussion (50%), team-based learning (47%), clickers (45%), simulation (25%), Interactive Web-based learning (19%) and process-oriented guided inquiry learning (12%). Promoters for active learning techniques include younger faculty age, lower faculty rank, and pharmacy practice or social/behavioral/administrative sciences departments. In the Middle Ease, Alruthia et al. surveyed faculty members in health colleges from 17 countries throughout the region [13]. The majority of pharmacy colleges respondents (*n* = 202) acknowledged utilizing various active learning strategies including class discussions (87%), small group discussions (56.9%), learning by teaching (49.5%), case studies and problem-based learning (41.1%). Several other studies have also described the incorporation of active learning techniques into pharmacy curriculum [12, 14–18].

Gamification or serious games have gained popularity recently [19–21]. In their overview of game implementation in the classroom, Biehle and Jeffres provided four essential fundamentals toward game development and use [21]. These fundamentals are, first, external motivation should be initiated to internally motivate the student to master the material. Second, students should benefit from the material whether they win or lose. Third, all students should equally be engaged in participating, and lastly, the objective of the game should be clearly explained to the students. Several systematic reviews concluded that gamification could improve the learning outcomes of health professions education, including pharmacy [22, 23]. The published literature includes descriptions of a lengthy list of the different types of games used to aid students in learning [17, 24–41]. The use of crossword puzzle (CWP) has been used occasionally in the literature with studies conducted in several pharmacy courses. Gaikwad et al. reported a significant improvement between CWP pre-and post-test scores when used in a pharmacology course covering antihypertensive and antiepileptic drugs for second-year Bachelor of Medicine and Bachelor of Surgery (MBBS) medical students [37]. Medical students in their fifth year of the MBBS degree indicated satisfaction with a CWP covering hormonal contraceptives [38]. Student perceptions toward the activity were also positive. Shah and colleagues developed a CWP during the pharmacology and medicinal chemistry module about anti-ulcer agents [36]. Over two years, post-activity surveys revealed that most students enjoyed the CWPs and felt as if it enhanced their learning.

Although it is assumed that active learning strategies will increase student engagement for most participants, there is scarce literature investigating whether there is an association between student learning styles and their preference for active learning strategies in the classroom. Conversely, when a mismatch occurs in the instructor’s teaching style and a student’s learning style, student learning may be impacted [42]. A study describing millennial students in the Philippines found that most students are application-oriented, focusing on applying what they studied to what they learned in practice [43]. This finding is important because modern pharmacy curricula include more application courses and experiential education. Crawford et al. compared the dominant learning styles of pharmacy students and faculty members and found no significant differences between faculty members and student learning styles [44]. However, the study found that learning styles differed by gender and faculty track (tenure vs. clinical).

Studies evaluating the CWP in pharmacy are still scarce. Additionally, none of the published work addressed pharmacy students’ learning style preferences and their perception of educational games. Moreover, the educational pathway for entry to practice as a pharmacist is different across the world [45]. In some countries, the Bachelor’s in pharmacy sciences (BPharm) is the only program to graduate pharmacists; in others like the United States, Doctor of Pharmacy (PharmD) is the sole program that graduates pharmacy practitioners. At the same time, some countries offer both programs (BPharm or PharmD) where graduates are licensed to practice pharmacy, such as the case in Saudi Arabia. The structure, educational level and focus of the BPharm and PharmD programs are different. In addition, each has its own set of competencies that suggest variations in teaching and learning approaches [46]. Hence for this study, we aimed to (1) evaluate the perception of BPharm and PharmD students toward the use of CWPs as an educational tool for enhancing their learning about anticoagulant therapeutics taught in the pharmacotherapy courses, (2) identify the preferred learning style among pharmacy students and (3) determine whether students’ learning style preferences, pharmacy program influenced their perception of this active learning technique.

## Methods

### Context and participants

 This is a cross-sectional study, where we used CWP as an active learning tool in therapeutic courses for students in the PharmD and BPharm programs. The PharmD program is a 6-year study plan (200 credit units) with considerable focus on pharmacotherapy (8 courses) and pharmacy practice lab (6 courses); these in total constitute (14%) of the curriculum. On the other hand, The Bachelor’s program is a 5-year study plan (173 credit units) considerably focused on pharmaceutical sciences, with only 6% of the credits going for pharmacotherapy (2 courses) and pharmacy skill labs (2 courses). Both programs are ACPE accredited, and faculty in the college teach in both curricula. However, the Bachelor’s program is delivered mainly by non-practicing faculty. The focus of this research is on therapeutic courses, PHCL 412 (PharmD course) and PHCL 416 (BPharm course), which start in the seventh level (third professional year). We targeted content related to the cardiovascular disease’s module, particularly anticoagulant therapeutics. The faculty covered the management of venous thromboembolism and atrial fibrillation with an overview of anticoagulant drugs over six didactic hours.

### Game objectives and development

**Fig. 1:**
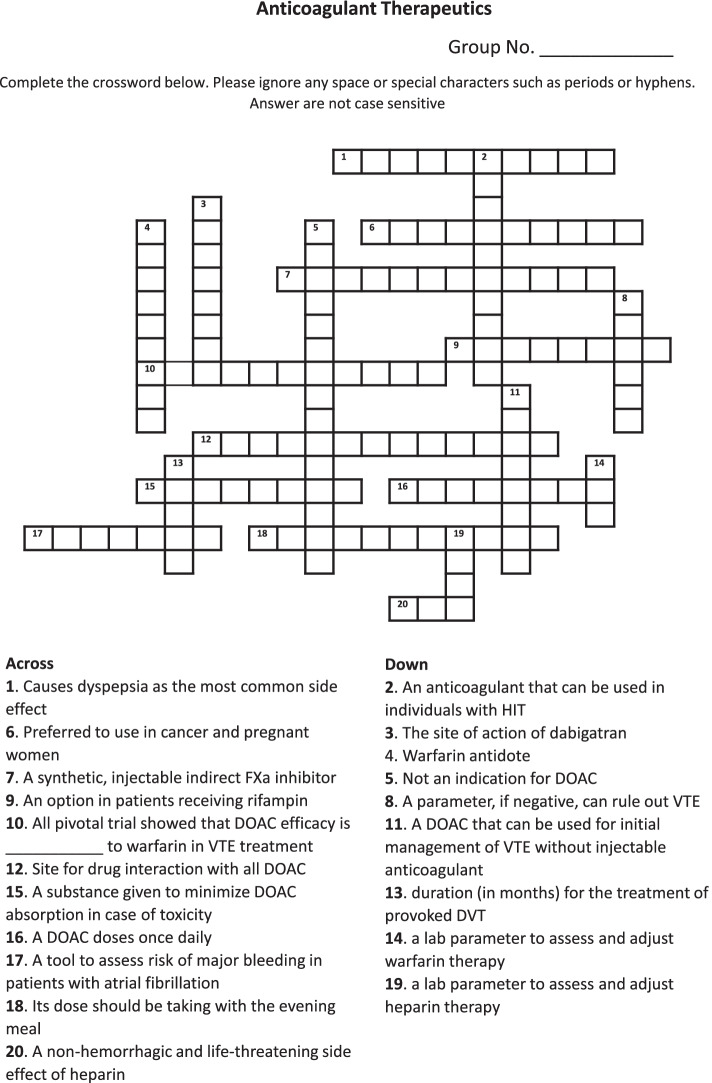
An Example of a CWP Clues

 The learning objectives related to anticoagulant therapy are presented in Table 1. CWP was created using a free online CWP generator (TheTeachersCorner.net). The course instructors developed the puzzle clues (questions and answers) based on the material covered during the lectures and designed to cover Bloom’s taxonomy of knowledge, comprehension, and application levels. The content validity of these CWPs was mapped to the intended learning outcomes of the modules and was peer-reviewed by another course instructor. Additionally, the CWP was piloted among a group of student interns in their advanced pharmacy practice experiential training. Printed copies of the CWP were provided to the students to solve immediately after the lecture (BPharm group, 10 clues CWP) or during the pharmacy practice lab (PharmD group, 20-clues CWP). Students were divided into groups, and 30 min was allocated for solving each CWP. Although students were expected to self-correct the answers to solve the puzzle, a debriefing session was provided to discuss any confusing points or misconceptions related to the topic. An example puzzle is shown in Fig. 1.

At the end of the activity, students were asked to complete two post-activity surveys administered via SurveyMonkey. The first survey was the Pharmacists’ Inventory of Learning Styles (PILS), a survey tool validated for pharmacy practice adapted from Kolb’s learning style inventory [47]. The instrument categorizes learning styles into four groups: accommodator, assimilator, converger and diverger. The second survey was an 8-item questionnaire to measure students’ attitudes towards the use of games in learning developed by Shah et al. [36]. Both surveys were anonymous; students were asked to complete the PILS survey first to identify their PLS and include it in a mandatory field in the perception survey. Students in the study consented to participate. Participation in the surveys was voluntary, and no extra marks were given to the students for completing the puzzle or the surveys. Students were also informed that their grades in the course would not be impacted by their participation, or lack thereof, in the activity. We implemented the CWP over three consecutive years using different puzzles.

**Table 1:** Specific Learning Objectives for Lectures Covering Anticoagulation therapeutics

PHCL 412 and PHCL 416 Anticoagulation Therapy Lectures Objectives:
1. Compare and contrast between the different anticoagulant therapies about:
a. Place of therapy
b. Mechanisms of action
c. Advantages and disadvantages in terms of their pharmacokinetic and pharmacodynamic properties (e.g. dosing, the onset of action, drug & food interactions, etc.)
d. *Key pivotal clinical trials of anticoagulant drugs
e. Side effects and management
f. Monitoring of drug therapy
g. Patient education
h. *Use in a selected population (e.g. cancer patients, pregnancy, morbid obesity, renal impairment, and pediatrics)
2. Apply evidence-based guideline recommendations for the use of anticoagulants in the:
a. Prevention and treatment of venous thromboembolism
b. Stroke prevention in Atrial fibrillation
c. ^a^Perioperative management of anticoagulant therapy

^a^ not included in the learning objectives for PHCL416. PHCL 412: is the course code for the therapeutic module offered in the Doctor of Pharmacy Program. PHCL 416: is the course code for the therapeutic module offered in the Bachelor of Pharmacy program, PHCL

### Statistical analysis

The data were analyzed using IBM SPSS Statistic version 22. Categorical data were presented as frequencies (N) and percentages (%), while continuous data were expressed by the mean and standard deviation (SD). A normality test (Kologrove-Smirnov and Shapiro-Wilk test) was conducted for the dependent variables (student perceptions of CWPs) and the independent variables (gender and education degree) to determine whether students’ perceptions were normally distributed among gender categories (i.e. male and female) and education degree categories (i.e. PharmD and BPharm). In the perception survey, we consolidated the data for the 5-Likert scale by combining the positive responses (strongly agree and agree) as one data set and the negative responses (strongly disagree and disagree) as another data set to facilitate statistical analysis. As data were not normally distributed, the Mann-Whitney U test was used to test for the differences in perception among the independent groups. We used ANOVA to compare between the survey questions means and the different learning styles. Responses to the open-ended question of the survey were analyzed and presented as perception themes based on keywords/phrases in the students’ responses. The reliability of the scale was assessed by Cronbach’s alpha value. A value of alpha ≥ 0.7 was considered acceptable reliability. The Cronbach’s Alpha for the instrument in this study was 0.881.

## Results

During the three consecutive years of the puzzle implementation, 380 students enrolled in PHCL 412 and 76 enrolled in PHCL 416 course. Data were available on 267 students who participated in the game (response rate of 59%). The mean age of the students was (21.34 ± 1.21), the majority were females (67%), and two-thirds (71%) were enrolled in the PharmD program. Table 2 summarizes the student demographics. On the PILS survey, the most preferred learning styles across all students were converger (*n* = 93, 35.6%) followed by assimilator (*n* = 66, 25.3%), while 41 students (15.7%) were multimodal. Learning style order of preference based on gender showed a significant difference (*p* < 0.05), with female students preferring assimilator and converger learning styles, while male students’ preference was higher for accommodator and diverger learning styles. Less than 10% of students could complete the puzzle in 30 min, and a few numbers had correct answers.

**Table 2:** Demographic characteristics (*N* = 267)

Characteristics	N(%)
Age (years)^a^	21.34 (1.21)
**Gender**	
Male	88 (33)
Female	179 (67)
**Learning style**^**b**^	
Accommodator	28 (10.7)
Assimilator	66 (25.3)
Converger	93 (35.6)
Diverger	34 (13.1)
Multimodal	41 (15.7)
**Educational degree**	
PharmD	191 (71.5)
BPharm	76 (28.5)

^a^mean(SD),^b^(6 students did not answer this question)

On the perception survey, items related to the value of CWP as a learning tool scored positively high by most students, 220 students (82.4%) perceived that CWPs helped them to identify the most important topics to focus on, 214 students (81.3%) perceived that the CWP served as a good review of the lecture material, 206 students (77.2%) thought CWP enhanced their learning, while 180 students (67.4%) believed they learned from the class because of the CWP. Majority of students (*n* = 194, 74.2%) perceived material on the CWP was pertinent to what they learned in class and that they enjoyed the classmate interaction while solving the puzzle (*n* = 211, 79%). Most of the students (*n* = 195, 73%) perceived the amount of time given was sufficient to solve the puzzle and expressed a preference to have extra credits for such activities (*n* = 196, 73.4%). Table 3 summarizes students’ perceptions of CWP as a learning tool.

**Table 3:** Student perceptions of CWPs as a study tool (*N* = 267)^a^

Perception Survey Items	Mean (SD)	Strongly Agree N (%)	Agree N (%)	Neutral N (%)	Disagree N (%)	Strongly Disagree N (%)
The CWP provided enhanced my learning	4.1(0.9)	87(32.6)	119(44.6)	45(16.9)	9(3.4)	7(2.6)
Enjoyed classmate interaction and reviewing material while solving the CWP.	4.1(0.9)	107(40.1)	104(39)	42(15.7)	9(3.4)	5(1.9)
CWP oriented us to the topics that we should focus on.	4.1(0.8)	99(37.1)	121(45.3)	35(13.1)	7(2.6)	5(1.9)
Length of time provided for solving the CWP was sufficient.	3.9(0.9)	95(35.6)	100(37.5)	49(18.4)	18(6.7)	5(1.9)
The material on the CWP was pertinent.	3.9(0.8)	80(30)	118(44.2)	61(22.8)	3(1.1)	5(1.9)
Learned more from the class because of the CWP.	3.8(0.9)	65(24.3)	115(43.1)	64(24)	18(6.7)	5(1.9)
Solving CWP is a good review of the material covered in the lecture.	4.0(0.8)	92(34.5)	125(46.8)	37(13.9)	9(3.4)	4(1.5)
Extra credit should be associated with activities such as a CWP.	4.0(0.9)	102(38.2)	94(35.2)	52(19.5)	13(4.9)	6(2.2)

^a^Survey adapted with permission from Ref [36]

There was a significant difference in students’ perception of CWPs among gender groups. Female students had a significantly higher mean score for the statement “length of time provided for solving the puzzles was sufficient” than their male counterparts (*p* = 0.001) (Table 4). Furthermore, there was a significant difference in student perceptions about CWPs among the two different undergraduate programs. The BPharm students’ mean score was significantly higher on several perception survey items compared to PharmD students, specifically on statement items “CWPs oriented them to the topics they should focus on” (*p* = 0.003), “the length of time provided for solving the puzzles was sufficient” (*p* = 0.001), and “the material on the puzzles was pertinent” (*p* = 0.038).

**Table 4:** Association of Student Perceptions of CWPs with demographic Characteristics

Perception Survey Items	Gender		*P*-value^a^	Education degree		*P-*value^a^
	**Male**	**Female**		**PharmD**	**BPharm**	
	**Mean (SD)**	**Mean (SD)**		**Mean (SD)**	**Mean (SD)**	
The CWP provided enhanced my learning	4.0(0.98)	3.9(0.90)	0.462	3.9(0.91)	4.0(0.97)	0.463
Enjoyed classmate interaction and reviewing material while solving the CWP.	4.0(0.94)	4.1(0.91)	0.416	4.1(0.91)	4.1(0.94)	0.840
CWP oriented us to the topics that we should focus on.	4.0(0.93)	4.1(0.84)	0.741	4.0(0.85)	4.3(0.90)	**0.003**
Length of time provided for solving the CWP was sufficient.	3.7(1.02)	4.1(0.95)	**0.001**	3.8(0.98)	4.2(0.96)	**0.001**
The material on the CWP was pertinent.	3.8(0.88)	4.0(0.85)	0.163	3.9(0.82)	4.1(0.95)	**0.038**
Learned more from the class because of the CWP.	3.7(1.01)	3.8(0.91)	0.668	3.7(0.91)	3.9(1.01)	0.086
Solving CWP is a good review of the material covered in the lecture.	4.1(0.83)	4.0(0.87)	0.971	4.0(0.86)	4.1(0.86)	0.146
Extra credit should be associated with activities such as a CWP.	4.1(0.87)	3.9(1.03)	0.051	3.9(0.99)	4.2(0.93)	**0.008**

^a ^Mann− Whitney U test

There was no association observed between learning style and students’ perception of CWP *(p* = 0.19). using the analysis of variance (ANOVA) to compare between the mean scores of the perception survey items and the PLS was also not significant. a significant association Table 5 summarizes the perceptions of CWPs across the different PLS.

**Table 5:** Students’ perceptions of CWPs across the preferred learning style

Perception Survey Items	Accommodator (*N* = 28)	Assimilator (*N* = 66)	Converger (*N* = 93)	Diverger (*N* = 34)	Multimodal (*N* = 41)	*P-value*^a^
The CWP provided enhanced my learning	4.00 (0.96)	4.03 (0.94)	4.04 (0.86)	4.18 (0.90)	3.85 (0.99)	0.661
Enjoyed classmate interaction and reviewing material while solving the CWP.	4.19 (0.68)	4.11 (1.05)	4.11 (0.84)	4.15 (0.99)	4.17 (1.00)	0.991
CWP oriented us to the topics that we should focus on.	3.89 (1.05)	4.32 (0.81)	4.05 (0.84)	4.18 (0.94)	4.07 (0.85)	0.192
Length of time provided for solving the CWP was sufficient.	3.74 (0.76)	4.08 (1.04)	3.99 (0.91)	4.12 (0.98)	3.88 (1.19)	0.512
The material on the CWP was pertinent.	3.74 (1.02)	4.15 (0.81)	3.99 (0.83)	4.12 (0.88)	3.73 (0.87)	0.063
Learned more from the class because of the CWP.	3.78 (0.97)	3.92 (0.97)	3.82 (0.88)	3.91 (1.00)	3.66 (0.91)	0.66
Solving CWP is a good review of the material covered in the lecture.	3.96 (0.98)	4.11 (0.96)	4.10 (0.79)	4.32 (0.73)	3.98 (0.88)	0.437
Extra credit should be associated with activities such as a CWP.	4.11 (0.80)	4.05 (1.09)	3.97 (1.03)	4.03 (0.94)	3.90 (0.92)	0.91

^a^Analysis of variance (ANOVA)

For the first open ended-questions at the end of the survey, 154 students provided feedback to the first question *“Please share any additional thoughts and feedback about using the game in the teaching-learning process (Feel free to write in Arabic or English)”*. We grouped the different responses and identified four types of perceptions. (1) Perceptions about how student’s felt about the game itself (*n* = 57) as indicated by words such as “fun”, “enjoyable”, “interesting”, “exciting”, “amazing” “good” “great” and “mind refreshing”. (2) Perceptions about the game effectiveness as tool to improve learning (*n* = 54) that was mainly captured by phrases such as “Break from the traditional teaching”, “helpful”, “information retention”, “review of learned knowledge”, “beneficial and creative”, “well organized” and “useful”. (3) Perceptions about the game as a self-assessment tool for learning (*n* = 22) through phrases such as “enhance memorization”, “focus on spelling skills”, “pay attention during class”, “aid recall”, “focus my learning”, “a way to understand” and “facilitated my learning”. (4) Perceptions about the learning environment (*n* = 12) with student’s using words such as “interactive”, “engaging”, “motivating”, “stimulating” and “competitive”.

For the second open ended question about *“What topic(s) in pharmacy curriculum you like to see taught in a game format?”*, there were 144 responses. Students suggested that CWP is an effective way to teach confusing subjects, or prior to exams and suggested to extend the use of CWP for other topics such as therapeutics (commonly mentioned topics: hypertension, atrial fibrillation, heart failure and ischemic heart disease), diabetes, antibiotics, drug-drug interactions, microbiology, medicinal chemistry, and several students suggested to use in all courses. The remaining of the comments were suggestion to improve future use of CWP. Students suggested to have shorter CWP clues, to give them enough time to prepare for the CWP, assign bonus grades, and use it as a pre-exam review. Table 6 summarize the comments shared by students under each perception theme.

**Table 6:** Themes of students’ free comments on the CWP activity

Feedback about using the game in the teaching-learning process (*N* = 154)	
**Perceptions about how student’s felt about the game itself** • “Very exciting we’d love more of this” • “I like the idea it is creative and fun” • “Exciting And enjoyable” • “I find it fun, and I truly enjoyed it” • “It was fun learning process solving the puzzle with my classmate” • “I liked how we did not use any reference to answer it was a mind refreshing activity.”	**Perceptions about the game effectiveness as tool to improve learning** • “it’s an effective method to enhance learning in fun way, it should be in every topic” • “I found the idea very different than our usual methods which is truly appreciated” • “It gives us a good review and it is not associated with marks” • “Solving crossword puzzles is good review of material I hope to get more for next subject” • “Interactive learning is fun and efficient way to obtain knowledge and should be used more in all classes rather than the traditional way of teaching” • “Change the narration style and create a stimulating learning environment for students” • “It is much better studying in game format rather than normal assignments.”
**Perceptions about the game as a self-assessment tool for learning** • “It makes me focus more on the spelling which is important” • “The activities enhance memorization of the information” • “Perfect method to evaluate our knowledge” • “It was fun and forces us to learn the spilling of drugs” • “Was great help to remember as reviewing after study “ • “Improves thinking and memorizing” • “I think it’s a good idea change in the routine it made me want to pay more attention in class” • “It facilities the learning and being familiar with the drugs”	**Perceptions about the learning environment** • “it’s good and make the class more active” • “It makes the group more comfortable with each other, so has a good process in communication” • “It is an amazing activity to be engaged in learning” • “It’s getting quite competitive” • “I had good time doing this game with my classmates and it makes me revise the information that I studied in therapeutic”

## Discussion

The pathophysiology and therapeutic of cardiovascular diseases, specifically topics about thromboembolism and atrial fibrillation are essential topics taught in the Pharm.D. and Bachelor programs in King Saud University, Riyadh, Saudi Arabia. These cardiovascular topics are usually introduced in the first therapeutic course series, and they require students to use critical thinking and clinical decision-making skills when implementing guideline recommendations, which most student perceive it to be challenging. The courses also rely on student’s prior knowledge on the pharmacology of cardiovascular drugs. Feedback from students and course faculty showed students’ knowledge about anticoagulant drugs pharmacology was suboptimal due to misalignment of some topics in the curriculum. To ease out student’s trepidation, the course instructors utilized variety of active learning pedagogy to engage students in this first therapeutic course, including CWP activity. To the best of our knowledge, this is the first study conducted to evaluate the pharmacy’ students’ perceptions about the use of CWP for enhanced learning about the anticoagulating therapeutics and their preferences of learning styles in Saudi Arabia.

In the present study, student perceptions about the value of CWP was generally positive. Overall, most of the students agreed that CWP enhanced their learning, oriented them to key information they need to focus on, improved retention of information and was a good review for the material covered in the lectures. These findings in consistent with other published studies [48–50]. Shah et al. reported higher agreement on the same survey items compared to the present results. 90% of students indicated positive perceptions such as CWPs enhanced their learning, helped them focus on important topics in the lectures, and served as a good review of the lecture material [36]. Mirroring the results from other studies about the recreational and fun aspects of the puzzles, most students enjoyed the classroom interactions while solving the puzzle. They felt the puzzle was interactive, enjoyable, and encouraged a competitive environment within the classroom [38, 50–56].

In the current study, students from the BPharm program reported statistically significant positive perceptions compared to PharmD students specifically on the following items: “CWP oriented us to the topics we should focus on” and “the material on the puzzle was pertinent”. This difference could be explained by the fact that traditional teaching is more predominant in BPharm curriculum. Crawford et al. found significant differences between tenure track and clinical track learning styles among faculty at the University of Illinois at Chicago Pharmacy college [44]. The tenure track had a predominantly abstract sequential learning style that focuses on theoretical concepts and analytical approaches. The clinical faculty have a more concrete sequential learning style that focuses on application and practicality of information. The use of active learning is reported to be higher in clinical faculty members and faculty members in the social/behavioral/administrative sciences [12] In KSU, BPharm curriculum is focused predominantly on pharmaceutical sciences, with only two therapeutic courses.

On the perception item about “the length of time provided for solving the puzzle was sufficient”, students from the BPharm program have a significantly higher agreement than the PharmD students (*p* = 0.001). We attributed this difference to the shorter puzzle administered to the BPharm group (10 clues) compared to the PharmD group (20 clues). Nonetheless, different studies reported described variable clues numbers ranging from 12 to more than 50 and completion time from 5 to 60 min, and all showed favorable agreement that the allocated time was adequate regardless of the size of the puzzle or completion time [36, 38, 48, 51–53, 57–60]. Notably, in the current study male students were less satisfied with the time provided for solving the puzzles than female students. Two factors could have produced such difference. First, active learning, specifically games, are less utilized by male faculty. This is supported by a recent study that found that female faculty are more likely to implement active learning strategies in classroom teaching than male faculty [13]. Furthermore, faculty from different generations could be differ in their interaction, learning, and teaching skills regardless of gender [8]. Second, gender variation in learning styles could have affected their perceptions about the time. Although some studies have reported more men being diverger and women have an assimilator learning style, others did not find such differences [44, 61–63].

The present study found that the predominant learning style in our student population is consistent with the learning styles of pharmacists and pharmacy students reported in the literature [43, 44, 47, 61, 64–66]. The learning styles primarily reported with the pharmacy profession are assimilator and converger. Similarly, in this current study, the converger learning style was predominant in both male (35%) and females (36%). The second most prominent learning style was assimilator in female students (33%) and divergent in male students (26%). It is worth noting that male representation in this study is only one third of the sample studied. We did not find a significant difference between student perceptions about CWP and their learning styles. One possible reason is that games in general are designed to accommodate all learnings styles [67]. Indeed, the attributes of the two predominant learning styles in this study (converger and assimilator) is consistent with the high perception of students to CWP as a learning tool. Converger has highly competitive nature and enjoy problem solving, application and activities such CWP appeal to this learning style [48]. Assimilator, on the other side, prefers organization, highly attentive to detail, inductive reasoning and prefer to get feedback regarding their performance all of these attributes are accommodated in activities such as CWP. For the remaining PLS, the diverger’s imaginative ability, and capability to see things from different perspectives games also appeals to this learning style. Accommodator learning style likes active experiences and CWP appeal to their ability to problem solve, risk taking and immediate reaction to circumstances.

We did not explore the perception of the game and or the PLS on student’s academic performance due to logistic factors. However, studies on the relationship between pharmacy students learning styles and academic performance are scarce and with divergent conclusions. A study conducted among medical students in Thailand showed that students’ learning styles differed between pre-clinical and clinical years. Furthermore, the learning style is only associated with students’ academic achievement during the pre-clinical years, with the reflective learning style associated with high academic success [68, 69]. Another study found a similar correlation between reflective abilities and academic success in undergraduate pharmacy education [70]. With assimilator and converger being the most common learning styles among pharmacy profession, reflective learning process is associated with assimilator but not converger who process actively [71]. Several other studies did not find an association between learning styles and academic achievement [62].

Millennials (those born between 1981 and 1996), represented by students participating in the current study, could have different approaches to learning and different preferences of learning styles, hence, different perceptions towards the variable approaches of learning. Corrido et al. showed that based on Vermunt’s Inventory of Learning Styles (ILS), millennial Filipino pharmacy students have a predominantly application-directed learning style (i.e. those who try to discover relationships between things and apply what they learned); followed by a reproduction-directed learning style (i.e. those who memorize the learning content in order to be able to reproduce it in a test) [43, 72]. Eng et al. also concluded that learning styles inventory could influence the learning environment during experiential education of millennial pharmacy students [73].

The previous studies and others raise an important issue to consider for both educators as well as students about identifying learning styles to improve learning experience rather than to stigmatize. Student’s self-awareness about their learning styles can help them refine their study habits and encourage them to reach out to faculty members when struggling academically due differences in teaching and learning styles; therefore, the learning environment can be catered to maximize students’ gain, confidence, and ability to transfer learning. At the same time, for faculty and preceptors, self-awareness of their own learning style and that of their students could help improve teaching styles and promote better approaches to better facilitate teaching and assessment.

Although the study didn’t include a qualitative component, our analysis of the feedback response on the open-ended questions agrees with several literature that also identified similar predominant themes such as fun, engaging, and promoting understanding [51, 54]. One of the interesting comments repeated by several students is the fact that spelling was an obstacle while solving the puzzle. Other researchers reported similar spelling challenges surfaced while solving CWPs [50, 56, 74, 75]. For pharmacy students, such accuracy is vital to patient and medication safety. The positive reception toward this tool encourages faculty members to develop creative tool that carry low burden (such as the CWP) to engage students especially in large class-size, which can be deterrent to incorporating active learning in teaching [76].

This study had several limitations. First, due to logistic reasons, administering the puzzle in relation to the lecture delivery was variable among the cohorts (right after the lecture, during the same week, or two weeks later). Although this might have affected the recall of information, the several open comments about spelling difficulties could be the main reason for not getting the right answers. Secondly, there was only one BPharm cohort group compared to three PharmD cohort groups. Thirdly, the length of the puzzle used for the BPharm group was shorter (10 questions and clues vs. 20 for the PharmD group); this is mainly because the content material for the BPharm is less than the PharmD and the need to deliver the puzzle in the short break after the lecture immediately. The learned information could have still been fresh and easy to retrieve. Hence, the perception of time sufficiency was satisfactory. Fourthly, the preferred learning style of the faculty was not assessed to determine if the high student’s perception is influenced by the educator learning style. Lastly, we did not assess the impact of the game on short- or long-term retention of the information, nor on student’s final grade in the course or pre- and post-testing examination, or academic performance; however, the activity was purposely implemented to guide students towards the most important information about the topic and assist them to identify any gaps in knowledge or understanding.

### Conclusions

The study demonstrated that students valued active learning engagement in the class environment using the CWPs. CWPs presented an easy and versatile tool that can be customized in difficulty to enhance students’ educational experience and promote more self-awareness of the teaching and learning trends for both students and faculty. Providing students with a self-learning tool and engaging them during the learning process may lead to better understanding, retention, and application of the learned material and eventually in academic performance. Future studies should investigate the impact of CWPs on both short- and long-term retention of the lecture material.

## Data Availability

The datasets used and/or analysed during the current study are available from the corresponding author on reasonable request.

## Abbreviations

ACPE: Accreditation Council for Pharmacy Education
BPharm: Bachelor of Pharmacy
CWP: Crossword Puzzle
KSU: King Saud University
PharmD: Doctor of Pharmacy
PILS: Pharmacists’ Inventory of Learning

## References

[1] Hidayat LV, Kim S, Sullivan E, Salbu M (2012). Pharmacy student absenteeism and academic performance. Am J Pharm Educ..

[2] Stoner SC, Fincham JE (2012). Faculty role in classroom engagement and attendance. Am J Pharm Educ..

[3] Westrick SCH, McDonough KL, Breland SK (2009). Factors influencing pharmacy students’ attendance decisions in large lectures. Am J Pharm Educ..

[4] Hartley J, Cameron∗ A. Some observations on the efficiency of lecturing. Educational Review. 1967;20(1):30–37.

[5] Hartley J, Davies IK (1978). Note-taking: A critical review. Program Learn Educ Technol..

[6] Thomas EJ (1972). The Variation of Memory with Time for Information Appearing During a Lecture. Stud Adult Educ.

[7] Boomers OD, Gen-Xers (2003). Understanding the “New Students.”. Educ Rev..

[8] Elshami WS, Coumaravelou, Taha MHA, Mohamed Elhassan; Abuzaid, Mohamed; Al Kawas, Sausan. Bridging the Gap in Online Learning Anxiety: Generation X teaching Millennial and Z generations. Sultan Qaboos University Medical Journal [SQUMJ]. 2021. 10.18295/squmj.4.2021.040 PMC8631220 34888072

[9] Abdulmajed H, Park YS, Tekian A (2015). Assessment of educational games for health professions: a systematic review of trends and outcomes. Medical Teacher..

[10] Education ACoP. PharmD Program Accreditation. https://www.acpe-accredit.org/pharmd-program-accreditation/. Accessed 20 Mar 2021.

[11] Gleason BLP, Resman-Targoff MJ, Karr BH, McBane S, Kelley S, Thomas K, Denetclaw T (2011). An active-learning strategies primer for achieving ability-based educational outcomes. Am J Pharm Educ..

[12] Stewart DWB, Clavier SD, Wyatt CW (2011). Active-learning processes used in US pharmacy education. Am J Pharm Educ..

[13] AlRuthia YA, Alodaibi Solaiman, Almutairi Faris, Algasem Lama, Alrabiah Reem, Sales Haitham K, Alsobayel Ibrahim (2019). Ghawaa, Yazeed. The use of active learning strategies in healthcare colleges in the Middle East. BMC Med Educ..

[14] Aburahma MH, Mohamed HM (2015). Educational Games as a Teaching Tool in Pharmacy Curriculum. Am J Pharm Educ..

[15] Berger JB, De Mooij N, Sutter Widmer J, Szilas D, De Vriese N, Bugnon C (2018). An open randomized controlled study comparing an online text-based scenario and a serious game by Belgian and Swiss pharmacy students. Curr Pharm Teach Learn..

[16] Persky AM (2008). Multi-faceted approach to improve learning in pharmacokinetics. Am J Pharm Educ..

[17] Persky AM, Stegall-Zanation J, Dupuis RE (2007). Students perceptions of the incorporation of games into classroom instruction for basic and clinical pharmacokinetics. Am J Pharm Educ..

[18] Tatachar AL, Gibson Feiming, Kominski Caitlin M (2016). Carol. Pharmacy students’ perception of learning and satisfaction with various active learning exercises. Curr Pharm Teach Learn..

[19] Sera L, Wheeler E (2017). Game on: The gamification of the pharmacy classroom. Curr Pharm Teach Learn..

[20] Cain J, Piascik P (2015). Are Serious Games a Good Strategy for Pharmacy Education?. Am J Pharm Educ..

[21] Biehle L, Jeffres M (2018). Play games and score points with students. The clinical teacher..

[22] Van Gaalen ABJ, Schönrock-Adema J, Bouwkamp-Timmer T, Jaarsma ADC, Georgiadis JR. Gamification of health professions education: a systematic review. Adv Health Sci Educ. 2020:1–29. 10.1007/s10459-020-10000-3 PMC8041684 33128662

[23] Shawaqfeh MS. Gamification as a learning method in pharmacy education. J Pharma Care Health Sys. 2015;10(2). S2-004.

[24] Oliver CH, Beavers P, Gibbs M, Goeckner E, Miller K (1995). Experiential Learning About the Elderly: The Geriatric Medication Game. Am J Pharm Educ..

[25] Rose TM (2011). A board game to assist pharmacy students in learning metabolic pathways. Am J Pharm Educ..

[26] Smith CR, Blais P, Schneck M (2017). Evaluation of two different poverty simulations with professional phase pharmacy students. Curr Pharm Teach Learn..

[27] Clarke C, Sedlacek RK, Watson SB (2016). Impact of a Simulation Exercise on Pharmacy Student Attitude toward Poverty. Am J Pharm Educ..

[28] Ee RWX, Yap KZ, Yap KY (2018). Herbopolis - A mobile serious game to educate players on herbal medicines. Complement Ther Med..

[29] Plakogiannis RS, Hernandez A, Nogid N (2020). A heart failure themed escape room approach to enhance pharmacy student learning. Curr Pharm Teach Learn..

[30] Cain J (2019). Exploratory implementation of a blended format escape room in a large enrollment pharmacy management class. Curr Pharm Teach Learn..

[31] Clauson AH, Frame L, Hagan T, Bynum A, Thompson LA, Kiningham ME (2019). An innovative escape room activity to assess student readiness for advanced pharmacy practice experiences (APPEs). Curr Pharm Teach Learn..

[32] Eukel HN, Frenzel JE, Cernusca D (2017). Educational Gaming for Pharmacy Students - Design and Evaluation of a Diabetes-themed Escape Room. Am J Pharm Educ..

[33] Tietze KJ (2007). A bingo game motivates students to interact with course material. Am J Pharm Educ..

[34] Kennedy D, Fanning K, Thornton P (2004). The Age Game: An Interactive Tool to Supplement Course Material in a Geriatrics Elective. Am J Pharmaceut Educ..

[35] Chavez BG, Eric H, Pathak Rolee, Volino Lucio R (2012). Popular game shows as educational tools in the pharmacy classroom. Curr Pharm Teach Learn..

[36] Shah S, Lynch LM, Macias-Moriarity LZ (2010). Crossword puzzles as a tool to enhance learning about anti-ulcer agents. Am J Pharm Educ..

[37] Gaikwad N, Tankhiwale S (2012). Crossword puzzles: self-learning tool in pharmacology. Perspect Med Educ..

[38] Patrick SV, Giri K, Datta VP, Kumawat D, Singh P, Matreja P (2018). The usefulness of crossword puzzle as a self-learning tool in pharmacology. J. Adv. Med. Educ. Prof..

[39] Rahim ASA, Ziden AA, Yap BK (2020). Gamified Online Quizzes: Pharmacy Student Perceptions of Learning in an Undergraduate Medicinal Chemistry Course. Malays J Pharm..

[40] Frey KM (2020). Structure activity relationship (SAR) maps: A student-friendly tool to teach medicinal chemistry in integrated pharmacotherapy courses. Curr Pharm Teach Learn..

[41] Yuriev E, Capuano B, Short JL (2016). Crossword puzzles for chemistry education: learning goals beyond vocabulary. Chem Educ Res Pract..

[42] Romanelli F, Bird E, Ryan M (2009). Learning styles: a review of theory, application, and best practices. Am J Pharm Educ..

[43] Carrido DI, Ramirez R-LF (2020). Learning styles of millennial students at a pharmacy school in the Philippines. Pharm Educ..

[44] Crawford SY, Alhreish SK, Popovich NG (2012). Comparison of learning styles of pharmacy students and faculty members. Am J Pharmaceut Educ..

[45] Supapaan T, Low BY, Wongpoowarak P, Moolasarn S, Anderson C (2019). A transition from the BPharm to the PharmD degree in five selected countries. Pharm Pract.

[46] Arakawa N, Bruno-Tomé A, Bates I. A global comparison of initial pharmacy education curricula: an exploratory study. Innovations Pharm. 2020;11(1). 10.24926/iip.v11i1.2093 PMC8132530 34017634

[47] Austin Z. Learning styles of pharmacists: impact on career decisions, practice patterns and teaching method preferences. Pharm Educ. 2004;4(1).

[48] Mohan BN, Vinod; Gowda Shivaraj, Arvindakshan Rajeev (2018). Crossword puzzle: a tool for enhancing medical students’ learning in microbiology and immunology. Int J Res Med Sci.

[49] Zamani P, Diparva Haghighi S, Ravanbakhsh M (2021). The use of crossword puzzles as an educational tool. J. Adv. Med. Educ. Prof..

[50] Shawahna R, Jaber M (2020). Crossword puzzles improve learning of Palestinian nursing students about pharmacology of epilepsy: Results of a randomized controlled study. Epilepsy Behav..

[51] Mshayisa VV (2020). Students’ perceptions of Plickers and crossword puzzles in undergraduate studies. J. Food Sci. Educ..

[52] Gilani RN, Priyanka; Daigavane Pallavi, Bajaj Pavan, Mankar Nikhil, Vishnani Rozina (2020). Crossword puzzle: An effective self-learning modality for dental undergraduates. J Datta Meghe Inst Med Sci Univ..

[53] Malini M, Sudhir G, Narasimhaswamy K (2019). Crossword puzzle as a tool to enhance learning among students in a medical school. Natl J Physiol Pharm Pharmacol..

[54] Nazeer MS, Ahmed Razia, Asad Mohammad Muzammil, Sami Mohammad Rehan, Hattiwale Waqas, Sreekanth Haroon Rasheed (2018). Crossword puzzles as an active learning mode for student directed learning in anatomy teaching: Medical undergraduate perceptions. Int J Med Res Health Sci..

[55] Saxena AN, Raenelle; Pahwa Punam, Mills Sheryl (2009). Crossword puzzles: active learning in undergraduate pathology and medical education. Arch Pathol Lab Med..

[56] Crossman EK, Crossman SM (1983). The crossword puzzle as a teaching tool. Teach Psychol..

[57] Mueller ST, Veinott ES. Testing the effectiveness of crossword games on immediate and delayed memory for scientific vocabulary and concepts. Paper presented at: CogSci2018.

[58] Raines DA (2010). An innovation to facilitate student engagement and learning: Crossword puzzles in the classroom. Teach Learn Nurs..

[59] Rajyaguru KA, MN Mohd; Nadiawati, AR; Reddy, SC. An Innovative Crossword Puzzle Tool to Evaluate the Undergraduate Medical Student’s Knowledge in Forensic Medicine. J Adv Med Med Res. 2016:1–7.

[60] Saran R, Kumar S (2015). Use of crossword puzzle as a teaching aid to facilitate active learning in dental materials. Indian J Appl Res..

[61] John EMN, Ming Chin Fen, Hong Long Chiau, Hassan Yet Hoi (2016). Yahaya. Learning style preferences of undergraduate pharmacy students in a Malaysian public university. Indian J Pharmaceut Educ Res..

[62] Willis SCP, Mrs Harsha; Silkstone, Victoria; Austin, Zubin. Unpacking links between learning gains, learning styles and achievement amongst 1 st year pharmacy students at Manchester Pharmacy School: Social Pharmacy, Manchester Pharmacy School; 2015.

[63] Alonso-Martín PC-D, Granado-Alcón Rocío, Lago-Urbano Carmen (2021). Martínez-García, Concha. Variability of Higher Education Students’ Learning Styles Depending on Gender, Course, Degree and Institutional Context. Sustainability..

[64] Bell B, Koch J, Green B. Assessing Learning Styles of Pharmacy Students Using the VARK Questionnaire. 2014.

[65] Pungente MD, Wasan KM, Moffett C (2003). Using Learning Styles to Evaluate First-Year Pharmacy Students’ Preferences Toward Different Activities Associated with the Problem-Based Learning Approach. Am J Pharm Educ..

[66] Williams B, Brown T, Etherington J (2013). Learning style preferences of undergraduate pharmacy students. Curr Pharm Teach Learn..

[67] Becker K. Games and learning styles. 2005.

[68] Jiraporncharoen WA, C; Chockjamsai, M; Euathrongchit, J. Learning styles and academic achievement among undergraduate medical students in Thailand. J Educ Eval Health Prof. 2015;12. 10.3352/jeehp.2015.12.38 PMC4536339 26165948

[69] Kolb AD. Individual learning styles and the Massachusetts: Sloan School of Management. 1971.

[70] Tsingos C, Bosnic-Anticevich S, Smith L. Does a learning style preference for processing information through reflection impact on the academic performance of a cohort of undergraduate pharmacy students? Pharm Educ. 2015;15.

[71] Kolb AD. The Learning Style Inventory: Technical Manual. Boston 1971.

[72] Vermunt JD. Inventory of learning styles (ILS). Tilburg, The Netherlands: Tilburg University, Department of Educational Psychology. 1994.

[73] Eng M (2013). Experience applying the Pharmacist Learning Styles Inventory (PILS) to experiential clerkships as a preceptor: A reflection. Curr Pharm. Teach. Learn..

[74] Abuelo A, Castillo C, May SA (2016). Usefulness of crossword puzzles in helping first-year BVSc students learn veterinary terminology. J. Vet. Med. Educ..

[75] Kumar L, Bangera S, Thalenjeri P (2015). Introducing innovative crossword puzzles in undergraduate physiology teaching- learning process. Arch Med Health Sci..

[76] Oyler DRR, Piascik F, Cain P (2016). Practical Insights for the Pharmacist Educator on Student Engagement. Am J Pharm Educ..

